# A210 HEPATOLITHIASIS IS A FREQUENT AND PROGNOSTIC FINDING IN PATIENTS WITH PRIMARY SCLEROSING CHOLANGITIS

**DOI:** 10.1093/jcag/gwab049.209

**Published:** 2022-02-21

**Authors:** B Jubran, M Ismail, M Stein, D H Little, B Hansen, A Gulamhusein, G Hirschfield

**Affiliations:** 1 Gastroenterology and Hepatology, University of Toronto, Toronto, ON, Canada; 2 Gastroenterology, University of Toronto, Toronto, ON, Canada; 4 Toronto Centre for Liver Disease Francis Family Liver Clinic, Toronto, ON, Canada

## Abstract

**Aims:**

Intrahepatic biliary stones (hepatolithiasis) are not well characterised in patients with primary sclerosing cholangitis (PSC).

**Methods:**

Chart reviews were conducted on 302 patients with a histologic or radiographic diagnosis of PSC followed at the Toronto Centre for Liver Disease. Radiographic data were collected for patients between the years 2008–2018. Depending on frequency of testing, magnetic resonance imaging (MRI) and ultrasound (US) data was reviewed every 3–5 years. We assessed factors associated with hepatolithiasis based on sex, race, age and phenotype of PSC and inflammatory bowel disease (IBD). Qualitative radiographic findings on image report review, episodes of cholangitis, endoscopic retrograde cholangiopancreatography (ERCP) and occurrence of cholangiocarcinoma (CCA), death and transplant were documented. Data are reported with median and IQR and analysed using χ ^2^ and Mann-Whitney U tests.

**Results:**

302 patients were reviewed. The median time to follow-up, defined as from date of diagnosis to last clinic visit or to transplantation date, was 98 months (IQR = 87). The mean age at diagnosis was 38 (SD = 15.1) years; 54% of patients were male. A total of 224 patients had IBD (74%). Of the 302 patients, 80 patients (26%) had evidence of hepatolithiasis on US or MRI. Patients with hepatolithiasis were more likely to be younger (37.4 vs 39.1, p = 0.025), male (65% vs. 50%, p = 0.021), and have large duct disease (99% vs. 88%, p = 0.004). Imaging report review revealed patients with hepatolithiasis were more likely to have intrahepatic biliary thickening (76% vs. 45%, p < 0.001), extrahepatic biliary thickening (69% vs. 50%, p = 0.003), focal biliary dilation (96% vs. 78%, p < 0.001) and disease characterised by more reported strictures on qualitative imaging report review (89% vs 69%, p < 0.001). Concomitant presence of cholelithiasis was greater in the hepatolithiasis vs. the non-hepatolithiasis group (45% vs. 19%, p < 0.001). There was no significant difference in the prevalence of hepatic or portal venous thrombosis in both groups. Patients with hepatolithiasis more likely have experienced acute ascending cholangitis (50% vs. 20%, p < 0.001) and need for ERCP (50% vs. 35%, p = 0.020). CCA was numerically higher in the hepatolithiasis group (8.75% vs. 4%, p = 0.1). Patients with hepatolithiasis received transplant more frequently (26.3% vs 12.2%, p < 0.001) with no significant difference in mortality.

**Conclusions:**

Hepatolithiasis is common in PSC and associated with an increased clinical and radiologic disease burden.

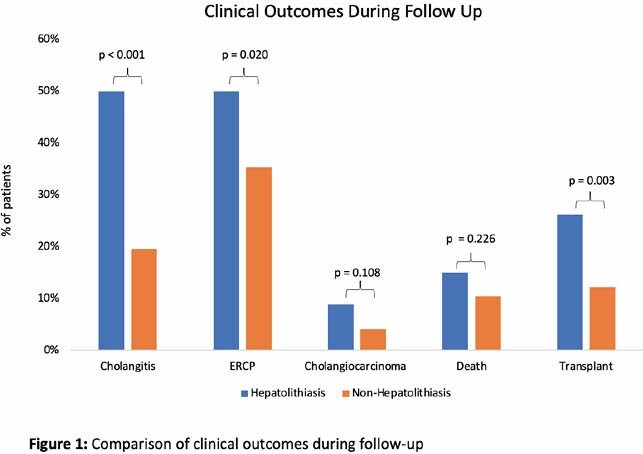

**Funding Agencies:**

None

